# Modulated Expression of Genes Encoding Estrogen Metabolizing Enzymes by G1-Phase Cyclin-Dependent Kinases 6 and 4 in Human Breast Cancer Cells

**DOI:** 10.1371/journal.pone.0097448

**Published:** 2014-05-21

**Authors:** Yi Jia, Joanne Domenico, Christina Swasey, Meiqin Wang, Erwin W. Gelfand, Joseph J. Lucas

**Affiliations:** Division of Cell Biology, Department of Pediatrics, National Jewish Health, Denver, Colorado, United States of America; Roswell Park Cancer Institute, United States of America

## Abstract

G1-phase cell cycle defects, such as alterations in cyclin D1 or cyclin-dependent kinase (cdk) levels, are seen in most tumors. For example, increased cyclin D1 and decreased cdk6 levels are seen in many human breast tumors. Overexpression of cdk6 in breast tumor cells in culture has been shown to suppress proliferation, unlike the growth stimulating effects of its close homolog, cdk4. In addition to directly affecting proliferation, alterations in cdk6 or cdk4 levels in breast tumor cells also differentially influence levels of numerous steroid metabolic enzymes (SMEs), including those involved in estrogen metabolism. Overexpression of cdk6 in tumor cell lines having low cdk6 resulted in decreased levels of mRNAs encoding aldo-keto reductase (AKR)1C1, AKR1C2 and AKR1C3, which are hydroxysteroid dehydrogenases (HSDs) involved in steroid hormone metabolism. In contrast, increasing cdk4 dramatically increased these transcript levels, especially those encoding AKR1C3, an enzyme that converts estrone to 17β-estradiol, a change that could result in a pro-estrogenic state favoring tumor growth. Effects on other estrogen metabolizing enzymes, including cytochrome P450 (CYP) 19 aromatase, 17β-HSD2, and CYP1B1 transcripts, were also observed. Interactions of cdk6 and cdk4, but not cyclin D1, with the promoter region of a cdk-regulated gene, 17β-HSD2, were detected. The results uncover a previously unsuspected link between the cell cycle and hormone metabolism and differential roles for cdk6 and cdk4 in a novel mechanism for pre-receptor control of steroid hormone action, with important implications for the origin and treatment of steroid hormone-dependent cancers.

## Introduction

Nearly all tumors, including those of the breast, have some defect in the network of cell cycle regulatory molecules (the cyclins, cyclin-dependent kinases [cdks] and cyclin-dependent kinase inhibitory proteins [CDKIs]) that control G1-phase entry and transit through progressive phosphorylation of pRb and its homologs [1]–[3]. Observations that many of the cdks have alternative substrates and functions and may in fact be dispensable for growth [4]–[6] raise the possibility that defects in their levels and regulation could affect the initiation and progression of tumors through alternative mechanisms.

The early G1-phase kinases cdk6 and cdk4, which are highly related structurally and are regulated through interactions with the same D-type cyclins and CDKIs [6], have been generally thought to play homologous functions in cells, with some notable exceptions [7], [8]. As expected, overexpression of cdk4, cdk6, or their regulatory D-type cyclins in cultured cells often leads to accelerated cell growth and their dysregulated function has been observed in many forms of cancer [2], [3], [9], [10]. However, it has also been observed that ectopically increasing cdk6 expression *reduced* proliferation of certain cell types, including mouse 3T3 fibroblasts and human breast tumor cell lines, through mechanisms involving p53 and/or p130/Rb2 [11], [12]. Furthermore, although overexpression or dysregulated function of cdk6 has been implicated in several types of cancer, including lymphoid malignancies [13], squamous cell carcinomas [14], and neuroblastomas [15], levels of cdk6 are *decreased* in many breast tumors and most breast tumor-derived cell lines [12], [16]. Ectopic expression of parkin in breast tumor cells also decreased their proliferation rate, with a concomitant *increase* in cdk6 levels [17]. Reduced cdk6 levels have also been observed in some pancreatic endocrine tumors as compared to normal tissue [18] and cdk6 overexpression resulted in decreased skin tumor development in a transgenic mouse model [19]. It has been reported that overexpression of cdk6 and cyclin D1 in chondrocytes, rather than enhancing proliferation, inhibited chondrocyte maturation and resulted in p53-dependent apoptosis [20].

To understand further the role of cdk6 and of its homolog cdk4 in breast cancer, their levels were increased by transfection in several breast tumor cell lines and effects on expression of genes encoding steroid metabolic enzymes (SMEs) were monitored. The expression of numerous SME genes was significantly altered, including those encoding CYP19 aromatase, AKR1C1, AKR1C3, 17β-HSD2 [21]–[24], and in normal human mammary epithelial cells (HMECs) overexpressing cdk4, CYP1B1 [25], [26]. Many of these enzymes and/or transcripts are altered in some fraction of breast tumors [26]–[28]. These findings are relevant to understanding the progression and treatment of hormone-dependent breast tumors, since many of them will have altered levels of G1-phase cell cycle regulatory proteins [1]–[3], [12]. Taken together, the results suggest a novel mechanism for pre-receptor control of steroid hormone action in breast tissue, in which cell cycle regulatory proteins modulate steroid hormone levels.

## Materials and Methods

### Cell Culture

MDA-MB-468 (cat #HTB-132), MDA-MB-453 (#HTB-131), and MCF-7 (#HTB-22) cells were obtained from the American Type Culture Collection (ATCC) and grown as described [12]. Cell lines were authenticated by the ATCC by DNA (STR) profiling and isoenzyme analysis. Early passage cells were stored in liquid nitrogen. After cells were thawed and placed into culture, they were grown and expanded in number for 1 week before being used in experiments. Experiments were conducted with cells that were passaged from 1 week to up to 6 months in continuous culture, after which new cell cultures were started. Cells showed little variation in apparent morphology or growth rate during this 6-month period of culture. Normal HMECs (#CC-2551) were from Lonza/Clonetics (Allendale, NJ) and maintained as described previously [12].

### Cell Transfection

Cdk6 (wild-type (WT) and dominant-negative (DN) forms) cdk4 and cyclin D1 cDNA sequences were in pCMV-vectors with a selectable G418 (Geneticin) marker [12]. The pCMV-cyclin D1 plasmid (cat #19927) was obtained from Addgene (Cambridge, MA). Transfection of cell lines and HMECs was performed as described previously [12] and in File S1.

### RNA Preparation and Analyses

RNA was prepared and RT-PCR and qRT-PCR were performed as previously [29], [30]. RT-PCR primer sequences were described previously [31], [32] and are listed in Table S1. Gene expression was analyzed by quantitative real-time PCR (qRT-PCR) as described previously [29]. TaqMan Gene Expression Assays (Applied Biosystems, Grand Island, NY) were used for AKR1C1 (ID #Hs04230636_sH), AKR1C3 (Hs00366267_m1), 17β-HSD1 (Hs00166219_g1), 17β-HSD2 (Hs00157993_m1), CYP1B1 (Hs00164383_m1), CYP19 (Hs00903411_m1), and GAPDH (Hs99999905_m1). For AKR1C2, custom primers and probe were made with sequences shown in Table S2. Real-time reactions were performed using an ABI 7700 Sequence Detection System (Applied Biosystems). Fold-changes in transcript levels were determined using the 2^−ΔΔ*C*T^ method, with normalization to expression of GAPDH [29]. Results were expressed as the mean±SEM. Student’s two-tailed *t* test was used to determine the level of difference between two groups. The *p* value for significance is designated as *p<0.05 and **p<0.01.

### Immunoblot Analysis

Cell extracts were prepared and resolved by polyacrylamide gel electrophoresis as described previously [12], [29]. Nuclear proteins were isolated using a NE-PER Extraction Kit (Pierce Chemical Co. #78833). Protein levels were compared in samples obtained from equal cell numbers and β-actin and HDAC2 were used as loading controls for whole cell and nuclear extracts, respectively. Reagents for chemiluminescence immunoblotting detection were from PerkinElmer Inc. (Waltham, MA). Antibodies used in the experiments are listed and described in Table S3A.

### Immunohistochemistry

Cell lines were grown in 8-well Millicell EZ slides (EMD Millipore, Billerica, MA) and processed for immunohistochemistry as described previously [12], with modifications described in File S1. Antibodies used are listed in Table S3B.

### Oligonucleotide “Pull-down” Assays

A well-characterized AP-1 binding site in the 17β-HSD2 gene [33] was synthesized (5′TCCAGTTAGTCATCGCTCCA), coupled to biotin, and used in “pull-down” experiments with nuclear extracts from parental or transfectant lines. Nuclear extracts were prepared using the NE-PER Kit (Pierce Chemical Co. #78833). Descriptions for isolation and analysis of protein-oligonucleotide complexes are in File S1.

## Results

### Overexpression of cdk6 in Breast Tumor Epithelial Cells Alters the Pattern of SME Gene Expression

**Figure 1:**
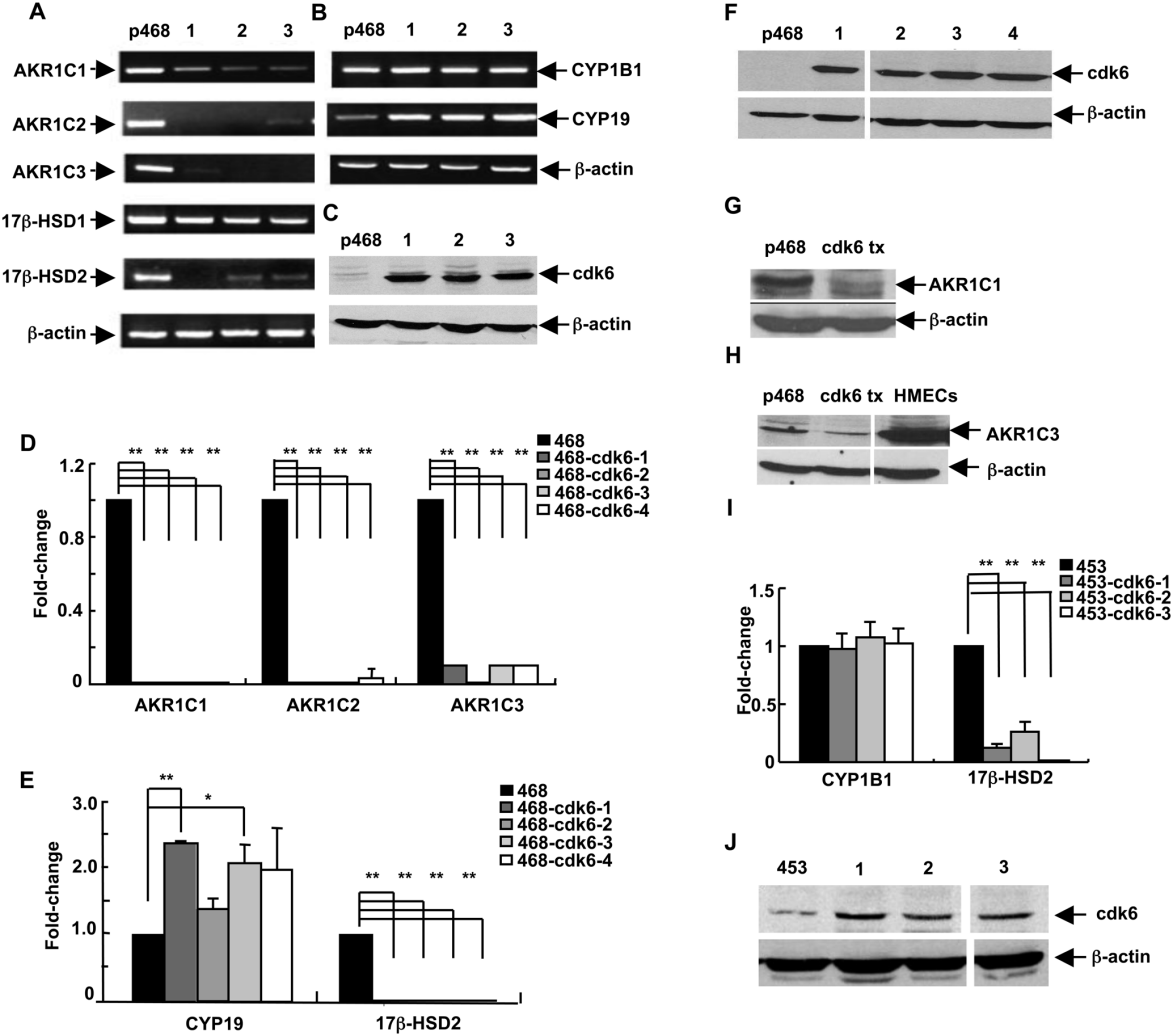
The pattern of SME gene transcripts in breast cancer cells is altered in stably-transfected cell lines with increased cdk6 protein levels. For panels (**A**) through (**H**), MDA-MB-468 cells were stably-transfected with a sequence encoding cdk6. (**A**) Levels of the 6 indicated transcripts were detected by RT-PCR in samples from parental MDA-MB-468 cells (“p468”) and in 3 stably-transfected clonal cell lines (labeled 1, 2, and 3). (**B**) Levels of the 3 indicated transcripts were detected by RT-PCR in the same cell lines. (**C**) Cdk6 protein levels were detected by immunoblot analysis, with β-actin levels as the loading control. (**D**) AKR1C1, AKR1C2, and AKR1C3 transcript levels in parental MDA-MB-468 cells (468) and in 4 stably-transfected cell lines (468-cdk6-1 through 468-cdk6-4) were detected and quantitated by qRT-PCR. (**E**) CYP19 and 17β-HSD2 transcript levels were quantitated in the 5 cell lines. (**F**) Cdk6 protein levels in parental MDA-MB-468 and the 4 cdk6-transfectant cell lines analyzed in (**D**) and (**E**) were detected by immunoblot analysis, with β-actin levels as the loading control. (**G**) AKR1C1 protein levels in parental MDA-MB-468 (p468) cells and a cdk6-transfectant cell line (cdk6 tx) were detected by immunoblot analysis, with β-actin as the loading control. (**H**) AKR1C3 protein levels in parental MDA-MB-468 cells (p468), in a cdk6 transfectant cell line (cdk6 tx) and in normal HMECs were detected by immunoblot analysis, with β-actin as the loading control. For panels (**I**) and (**J**), MDA-MB-453 breast epithelial cells were stably-transfected with sequences encoding cdk6. (**I**) CYP1B1 and 17β-HSD transcript levels in parental MDA-MB-453 cells (453) and in 3 stably-transfected cell lines (453-cdk6-1 through-4) were detected and quantitated by qRT-PCR. (J) Cdk6 protein levels in parental MDA-MB-453 and the 3 cdk6-transfectant cell lines analyzed in I were detected by immunoblot analysis, with β-actin levels as loading control. For panels (**D**), (**E**), and (**I**), the data are expressed as the mean±SEM, n = 3 times/group; *p<0.05 and **p<0.01. Note the differences in Y-axis scales on the graphs for panels (**D**), (**E**), and (**I**).

Many breast tumor cell lines have low or undetectable levels of cdk6 and overexpression of cdk6 by transfection reduces their rate of growth [12]. To characterize further the changes resulting from stable enhanced cdk6 expression, genes whose expression was altered after transfection of MDA-MB-468 breast tumor epithelial cells with cdk6 were examined by genome-wide transcriptional profiling. The gene whose level of expression was most dramatically altered was AKR1C1 (data not shown). This change in expression level was confirmed by RT-PCR and extended to examination of other transcript-encoding enzymes involved in steroid hormone metabolism. As shown in Figure 1, levels of transcripts for several genes encoding SMEs were dramatically altered in cdk6-transfectant cell lines. Analysis of 3 independently-isolated clonal lines stably expressing cdk6 showed that AKR1C1, AKR1C2, AKR1C3, and 17β-HSD2 transcript levels were markedly decreased as compared to levels in non-transfected cells whereas 17β-HSD1 transcript levels remained relatively unchanged by cdk6 expression (Figure 1A). Levels of CYP19 transcripts were increased but CYP1B1 levels were unchanged (Figure 1B). Levels of cdk6 protein in the transfected cell lines are shown in Figure 1C.

These observations were quantitated by qRT-PCR for parental MDA-MB-468 cells and 4 cdk6-transfected clonally isolated cell lines (Figures 1D and 1E). All 4 lines showed significant decreases in AKR1C1, AKR1C2, AKR1C3, and 17β-HSD2 transcript levels. CYP19 transcripts were increased approximately 2-fold in cdk6-transfectants. CYP1B1 transcript levels showed no consistent changes across the set of transfectant lines compared to the parental line with no significant changes on average (data not shown). Levels of cdk6 protein in these transfectant lines are shown in Figure 1F. Cultures of MDA-MB-468 cells and 2 cdk6-expressing lines were grown and harvested at various times, separated by several weeks of growth. Duplicate cultures showed a high degree of reproducibility and the changes in SME expression described above were validated (Figure S1).

It was noted that different SME genes were expressed at much different basal levels in breast tumor-derived cell lines. Cycle threshold (Ct) values suggested that AKR1C1, CYP1B1, and 17β-HSD2 were most robustly expressed in MDA-MB-468 cells (Table S4), followed by AKR1C3, which is the predominant AKR1C-family transcript in normal breast tissue [34]. Although 17β-HSD1 transcripts were detectable in the cells (Figure 1A) and did not change after transfection, they were at such low levels that these transcripts were excluded from further qRT-PCR analysis. Representative Ct values obtained using MDA-MB-468 transfectant cell lines are shown in Table S4. Examination of representative SME protein amounts showed clear decreases in AKRIC1 and AKR1C3 protein levels in cdk6-transfectants (Figures 1G and 1H), indicating that protein, and not only RNA, levels were altered. For comparison, the level of AKR1C3 protein is shown in normal HMEC cells (Figure 1H).

To determine the generality of the findings, clonally-derived cell lines with increased expression of cdk6 were also prepared using MDA-MB-453 cells. Analysis of SME gene expression showed that MDA-MB-453 cells had little or no transcripts for several of the genes analyzed above. Only CYP1B1 and 17β-HSD2 were expressed at robust levels (Table S5). In agreement with findings for MDA-MB-468 cells, 17β-HSD2 transcripts were greatly reduced in 3 MDA-MB-453-derived cdk6-expressing cell lines and CYP1B1 transcript levels were unchanged (Figure 1I). Levels of cdk6 protein in the lines are shown in Figure 1J. Table S5 demonstrates that levels of expression of SME genes in MDA-MB-468 cells were very similar to that seen in normal breast epithelial cells in culture. Both MDA-MB-453 and MCF-7 tumor cells showed greatly reduced expression of many of the SME genes. The results suggest that MD-MB-468 cells may be a good model system for studying SME gene expression and function.

**Figure 2:**
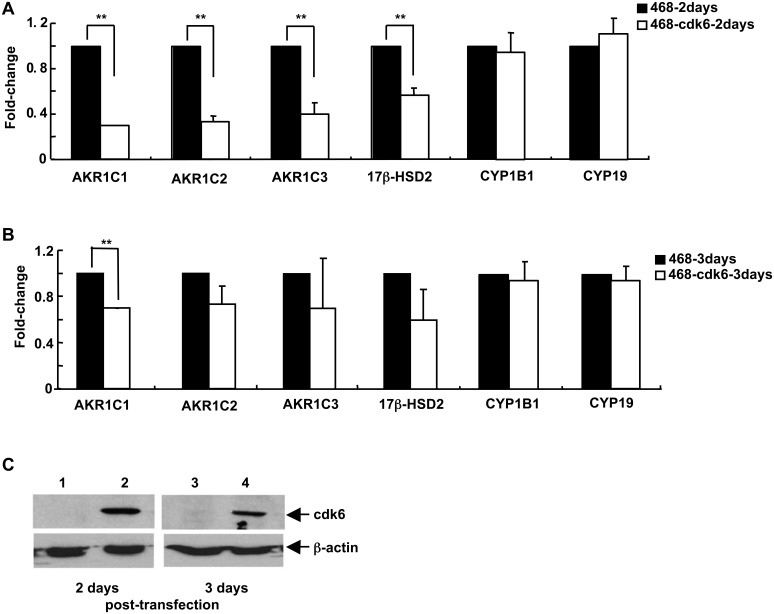
The pattern of SME gene transcripts in MDA-MB-468 breast cancer cells is altered by transient increased expression of cdk6. MDA-MB-468 cells were transiently transfected with a sequence encoding cdk6. RNA was prepared from cells transfected with the cdk6 sequence at (**A**) 2 days and (**B**) 3 days after transfection and assayed by qRT-PCR for transcript levels of AKR1C1, AKR1C2, AKR1C3, 17β-HSD2, CYP1B1, and CYP19. (**C**) The levels of cdk6 protein were determined in mock-transfected (lanes 1 and 3) and cdk6-transfected (lanes 2 and 4) cells at 2 days (lanes 1 and 2) and 3 days (lanes 3 and 4) after transfection, with β-actin as the loading control. For panels (**A**) and (**B**), the data are expressed as the mean ± SEM, n = 3 times/group and **p<0.01.

Transcription patterns in MDA-MB-468 cells were also examined after transient transfection. To assess transfection efficiency, cells were transfected using a plasmid encoding green fluorescent protein (GFP). Flow cytometric analysis indicated that about 45% of the cells were transfected (Figure S2A). MDA-MB-468 cells were transfected with a plasmid encoding cdk6 and assessed for levels of expression of 6 SME genes. Significant decreases in levels of all 4 transcripts that were reduced in stable transfectants (AKR1C1, AKR1C2, AKR1C3, and 17β-HSD2) were seen 2 days after transfection (Figure 2A). By 3 days (Figure 2B), levels for the 4 genes were still decreased, but differences were no longer statistically significant, except for AKR1C1 transcripts. No significant changes in levels of CYP1B1 transcript levels, a gene that did not change in prior analyses, or CYP19 were seen. That no significant change in CYP19 was observed is not surprising, given the transfection efficiency and the relatively modest alterations in transcript levels seen for this gene in stable transfectants (Figure 1E). The levels of cdk6 at 2 and 3 days post-transfection were determined by immunoblot analysis (Figure 2C). Expression was high at 2 days and decreased by 3 days after transfection. The decrease in cdk6 levels with time may be due to an increase in the number of non-transfected cells in the culture as a result of a greater growth rate of non-transfected cells, enhanced death of successfully transfected cells, or alternatively, to decreased plasmid expression with time.

### Increased Expression of cdk4 Alters the Pattern of SME Gene Expression but Differently than cdk6

**Figure 3:**
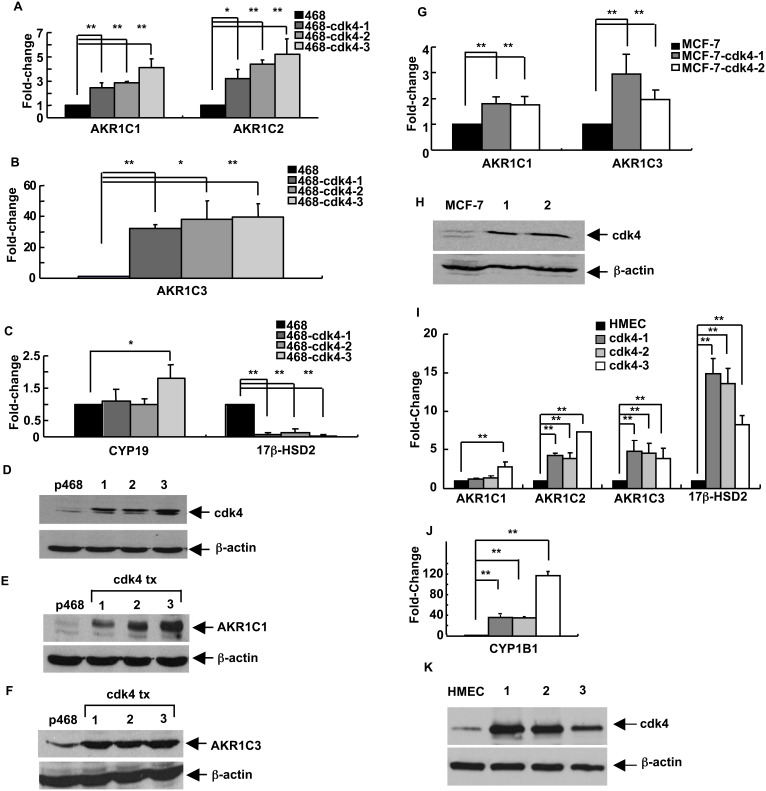
The pattern of SME gene transcripts in breast epithelial cells is altered by expression of cdk4. For panels (**A**) through (**F**), MDA-MB-468 breast epithelial cells were stably-transfected with a sequence encoding cdk4. (**A**) AKR1C1 and AKR1C2 transcript levels in parental MDA-MB-468 cells (468) and in 3 stably-transfected cell lines (468-cdk4-1 through 468-cdk4-3) were detected and quantitated by qRT-PCR. (**B**) AKR1C3 transcript levels were similarly quantitated in the 4 cell lines. (**C**) CYP19 and 17β-HSD2 levels were similarly quantitated in the 4 cell lines. (**D**) The cdk4 protein levels in parental cells (lane labeled p468) and the cdk4-transfectant cell lines (lanes labeled 1, 2, and 3) were detected by immunoblot analysis, with β-actin as loading control. Increased expression of both (**E**) AKR1C1 protein and (**F**) AKR1C3 protein in cells stably-transfected with the cdk4 sequence was demonstrated by immunoblot analysis, with β-actin as the loading control. For panels (**G**) and (**H**), MCF-7 breast epithelial cells were stably-transfected with a sequence encoding cdk4. (**G**) AKR1C1 and AKR1C3 transcript levels in parental MCF-7 cells and in 2 stably-transfected cell lines (MCF-7-cdk4-1 and MCF-7-cdk4-2) were detected and quantitated by qRT-PCR. (**H**) The cdk4 protein levels in parental cells (lane labeled MCF-7) and the cdk4-transfectant cell lines (lanes labeled 1 and 2) were detected by immunoblot analysis, with β-actin as the loading control. For panels (**I**) through (**K**), normal HMECs were transfected with a sequence encoding cdk4. (**I**) AKR1C1, AKR1C2, AKR1C3, and 17β-HSD2 transcript levels were quantitated in cells that had been transfected with cdk4, incubated for 48 hrs in regular medium, for 2 weeks in medium containing G418 and an additional week in medium without the selective agent. Results are shown for mock-transfected HMECs and 3 transfected cultures (cdk4-1 through cdk4-3). (**J**) CYP1B1 transcript levels were also determined in the cells. (**K**) Levels of cdk4 protein in the cultures were determined by immunoblot analysis, with β-actin as loading control. For panels (**A**), (**B**), (**C**), (**G**), (**I**), and (**J**), the data are expressed as the mean ± SEM, n = 3 times/group; *p<0.05 and **p<0.01. Note the differences in Y-axis scales on the graphs for panels (**A**), (**B**), (**C**), (**G**), (**I**), and (**J**).

Enhanced expression of cdk4 may play a role in breast cancer [3]. The effect of stable cdk4 overexpression in breast tumor cells was therefore examined. In comparison to cdk6 transfectants [11], [12], cdk4 overexpression caused an increased growth rate with MDA-MB-468 cells, as shown in Figure S3, and a pattern of changes in SME gene expression that differed markedly from that seen for cdk6 expression. Cdk4 overexpression resulted in *increases* in the levels of expression of AKR1C genes. AKR1C1 and AKR1C2 transcript levels were increased about 3- to 6-fold in 3 clonally-derived cdk4 overexpressing lines, as compared to the parental line (Figure 3A). A remarkable 30- to 50-fold increase in AKR1C3 transcript levels was seen in the cdk4-transfectants (Figure 3B). Cdk4 and cdk6 thus have opposing effects on expression of AKR1C-family transcripts. Cdk4 overexpression led to decreased levels of 17β-HSD2 transcripts (Figure 3C), as in cdk6 transfectants. No significant change in CYP19 levels were observed in 2 of the 3 clonal lines and a modest increase (less than 2-fold) was seen in 1 line. No significant change in CYP1B1 levels was observed (data not shown). Representative Ct values for the transcripts in parental MDA-MB-468 cells and cdk4-transfectant cell lines are shown in Table S4. Levels of expression of cdk4 in the transfectants and parental line are shown in Figure 3D. Analysis of protein levels for the AKR1C1 and AKR1C3 enzymes showed clear increases in the cdk4-overexpressing lines (Figures 3E and 3F).

Clonally-derived cell lines with increased expression of cdk4 were also prepared using the MCF-7 cell line. As shown in Table S5, MCF-7 cells display a fairly robust expression of the AKR1C1 and AKR1C3 genes. In agreement with the results for MDA-MB-468 cells, increased expression of the cdk4 protein resulted in increased levels of transcripts for these 2 genes, although the magnitude of the increases was less for this line (Figure 3G). Levels of cdk4 protein in the parental and transfectant lines are shown in Figure 3H. As indicated in Table S5, robust expression of the CYP1B1 gene was detected in the MCF-7 line, but no significant change in transcript levels was seen after transfection (data not shown).

The effect of enhanced expression of cdk4 in normal HMECs was also examined. Since these cells have a limited life span in culture, it was not possible to isolate stable clonally-derived lines expressing the kinase. Furthermore, although they can be transfected with an efficiency of about 48% (Figure S2B), expression of cdk4 or cdk6 in HMECs resulted in extensive cell death. However, through a modified protocol in which transfected cells were grown for 12–14 days in medium containing G418 to eliminate non-transfected cells and then for an additional week without the drug, sufficient cdk4-transfected cells for analysis could be obtained. As shown in Figures 3I–3K, significant, reproducible effects across a set of 3 transfectant cultures were observed. As in MDA-MB-468 and MCF-7 tumor cells, increases in the levels of AKR1C-family transcripts were seen in the cdk4-transfected normal breast cells (Figure 3I). Most notable, however, were dramatic increases in the levels of 17β-HSD transcripts (Figure 3I), which had decreased upon transfection of tumor cells, and very large increases of CYP1B1 (Figure 3J), which levels had not changed after transfection of tumor cells. The levels of cdk4 protein in the control and transfected cells are shown in Figure 3K. The results show that cdk4 levels altered SME gene expression in normal mammary epithelial cells, but with a different pattern than seen in tumor-derived cell lines. The results suggest that cdk-mediated regulation of SME levels may be a normal cellular mechanism that is subverted during tumorigenesis.

### Detection of Changes in cdk6, cdk4, and AKR1C3 Protein Amounts at the Cellular Level

**Figure 4:**
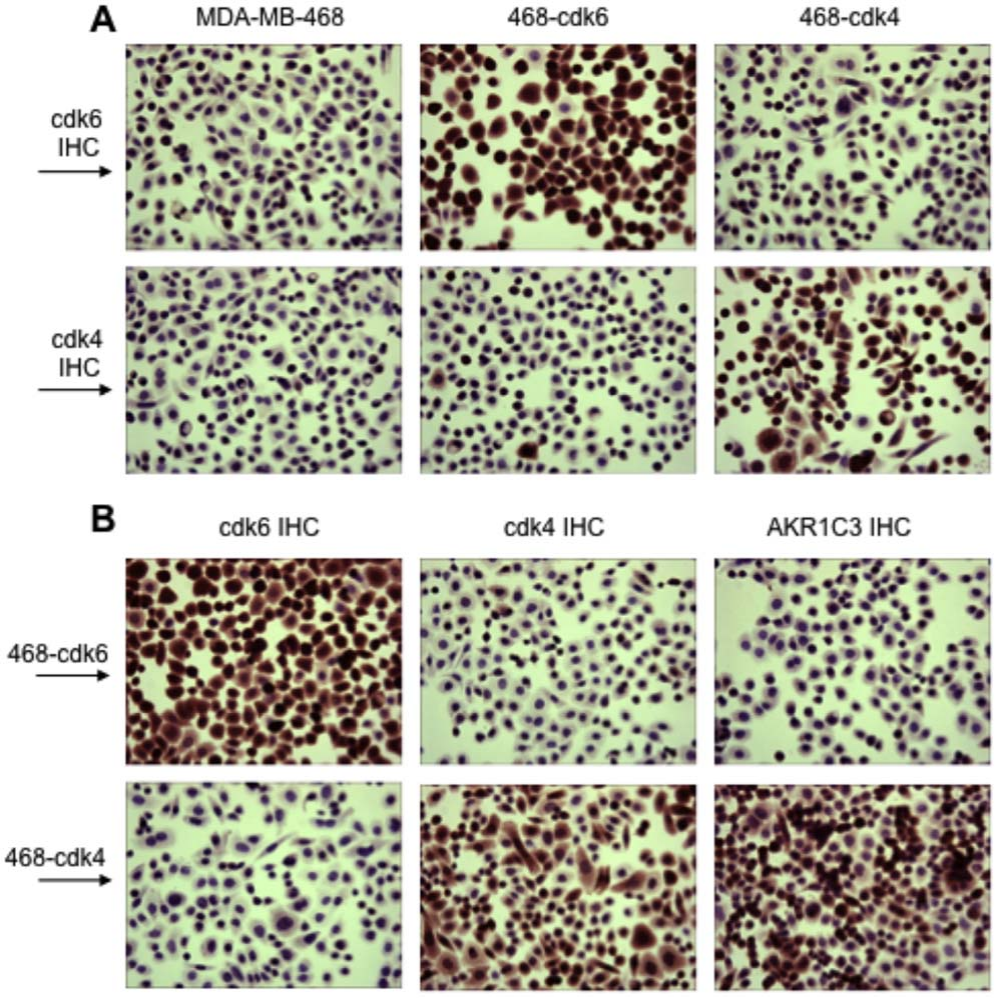
Immunohistochemical detection of cdk6, cdk4, and AKR1C3 in MDA-MB-468 cell lines transfected to express increased levels of cdk6 or cdk4. In the top row of panel (**A**), cdk6 was detected by immunohistochemistry (cdk6 IHC) in parental MDA-MB-468 cells and in cell lines transfected to express increased levels of either cdk6 (“468-cdk6” cells) or cdk4 (“468-cdk4” cells). In the second row, the 3 cell types were assessed for cdk4 levels. In the top row of panel (**B**), cells expressing increased amounts of cdk6 (“468-cdk6” cells) were analyzed by IHC for cdk6, cdk4, or AKR1C3. In the second row, cells expressing increased amounts of cdk4 (“468-cdk4” cells) were analyzed for the 3 molecules by IHC. Magnification: x200. The results indicate that nearly all of the cdk4-transfected cells have greatly increased levels of AKR1C3 protein.

In the experiments described above, changes in mRNA and protein amounts were detected in cell populations. Changes in protein amounts were also observed at the individual cellular level by immunohistochemistry. As shown in Figure 4A, MDA-MB-468 cells transfected with the sequence encoding cdk6 had more cdk6 protein than the parental cells, but there were little apparent changes in cdk4 levels. In contrast, cells transfected with the sequence encoding cdk4 had more cdk4 than parental cells, but there were little apparent changes in cdk6 levels. These findings were also substantiated by immunoblot analysis, as shown in Figure S4. Analysis of transfected cells supported the notion that increased cdk4-transfection resulted in a marked increase in AKR1C3 protein levels. As shown in Figure 4B, a large difference in cellular content of AKR1C3 was seen between cdk6 and cdk4 transfected cells, with increased AKR1C3 levels seen in virtually all cdk4-overexpressing cells.

### Increased Expression of Cyclin D1 does not Alter the Pattern of Expression of Most SME Genes

**Figure 5:**
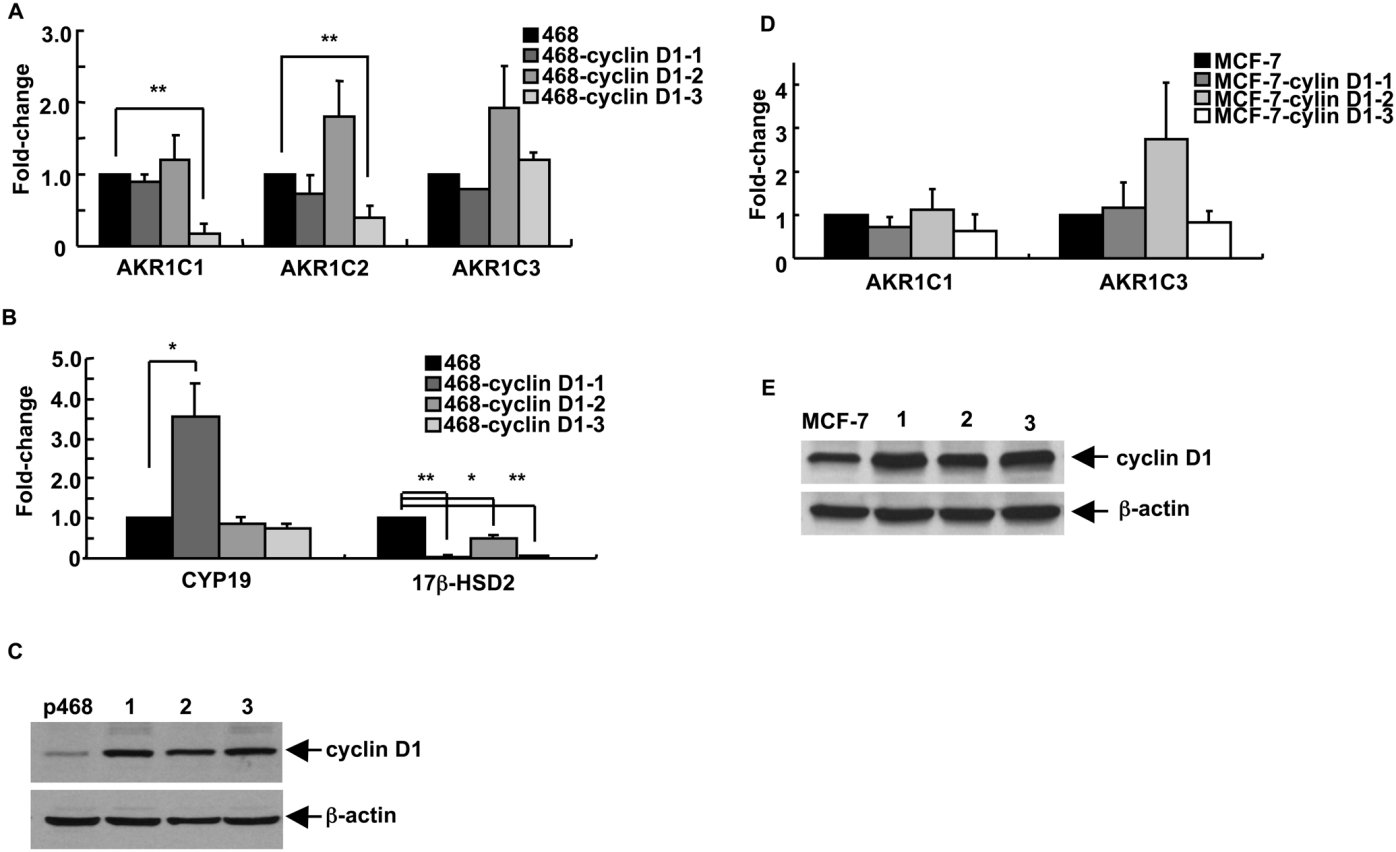
The pattern of SME gene transcripts in breast cancer cells is largely independent of cyclin D1 levels. For panels (**A**) through (**C**), MDA-MB-468 breast epithelial cells were stably-transfected with a sequence encoding cyclin D1. (**A**) AKR1C1, AKR1C2, and AKR1C3 transcript levels in parental MDA-MB-468 cells (468) and in 3 stably-transfected cell lines (468-cyclin D1-1 through 468-cyclin D1-3) were detected and quantitated by qRT-PCR. (**B**) CYP19 and 17β-HSD2 transcript levels were similarly quantitated in the 4 cell lines. (**C**) The cyclin D1 protein levels in parental MDA-MB-468 cells and the 3 cyclin D1-transfectant cell lines analyzed in panels (**A**) and (**B**) were detected by immunoblot analysis, with β-actin levels as the loading control. For panels (**D**) and (**E**), MCF-7 breast epithelial cells were stably-transfected with a sequence encoding cyclin D1. (**D**) AKR1C1 and AKR1C3 transcript levels in parental MCF-7 cells and in 3 stably-transfected cell lines (MCF-7-cyclin D1-1 through MCF-7-cyclin D1–3) were detected and quantitated by qRT-PCR. (**E**) The cyclin D1 protein levels in parental MCF-7 and the 3 cyclin D1-transfectant cell lines analyzed in panel (**D**) were detected by immunoblot analysis, with β-actin levels as the loading control. For panels (**A**), (**B**), and (**D**), the data are expressed as the mean ± SEM, n = 3 times/group; *p<0.05 and **p<0.01. Note the differences in Y-axis scales on the graphs for panels (**A**), (**B**), and (**D**).

Cdk4 and cdk6 form complexes with cyclin D-family proteins, an association necessary for enzymatic activity [6]. If changes in expression of SME genes induced by cdk6 and cdk4 were dependent on cdk/cyclin D1 enzymatic activity, alterations in cyclin D1 levels would likely have an impact on SME transcript profile. As shown in Figures 5A and 5B, overexpression of cyclin D1 had no consistent, reproducible effect on AKR1C1, AKR1C2, AKR1C3, or CYP19 transcript levels in MDA-MB-468 cyclin D1-transfectant lines. Some lines showed small increases or decreases in particular transcript levels but a consistent change across the set of lines was not observed. The lines did show significant decreases in 17β-HSD2 transcript levels. Cyclin D1 levels in the cell lines are shown in Figure 5C. Analysis of stably-transfected MCF-7 lines overexpressing cyclin D1 was also performed. In addition, this line showed no consistent, reproducible effects of cyclin D1 on SME gene expression (of AKR1C1 and AKR1C3) (Figure 5D). Levels of cyclin D1 protein in the cell lines are demonstrated in Figure 5E.

### Association of cdk6 or cdk4 with the Promoter Region of the 17β-HSD2 Gene

Cdks are known to have several roles beyond cell cycle regulation, some of which are transcriptional roles that appear to be independent of kinase function (reviewed in [35]). For example, both WT and DN forms of cdk10 bind to the Ets2 transcription factor and modulate its activity [36], [37]. We have previously shown that the suppression of cell growth mediated by cdk6 requires kinase activity [11], [12], but results obtained to date suggest that the effects of cdk6 upon SME gene transcription may indeed be independent of its kinase activity (and of cyclin D1, as indicated above). Thus, as shown in Figure S5, comparative RT-PCR analysis of parental MDA-MB-468 cells with cells stably-transfected with either WT or DN forms of cdk6 showed that either form of the cdk6 protein induced similar changes in the pattern of SME gene transcripts. Thus, expression of either the WT or DN forms of cdk6 effectively reduced AKR1C1 and AKR1C3 transcript levels; AKR1C2 levels were reduced in both types of transfectants, but more dramatically in the line expressing the WT form (Figure S5A). As shown in Figure S5B, levels of 17β-HSD2 transcripts were reduced in both WT- and DN-cdk6 transfectants and 17β-HSD1 transcripts were not altered in either cell line.

**Figure 6:**
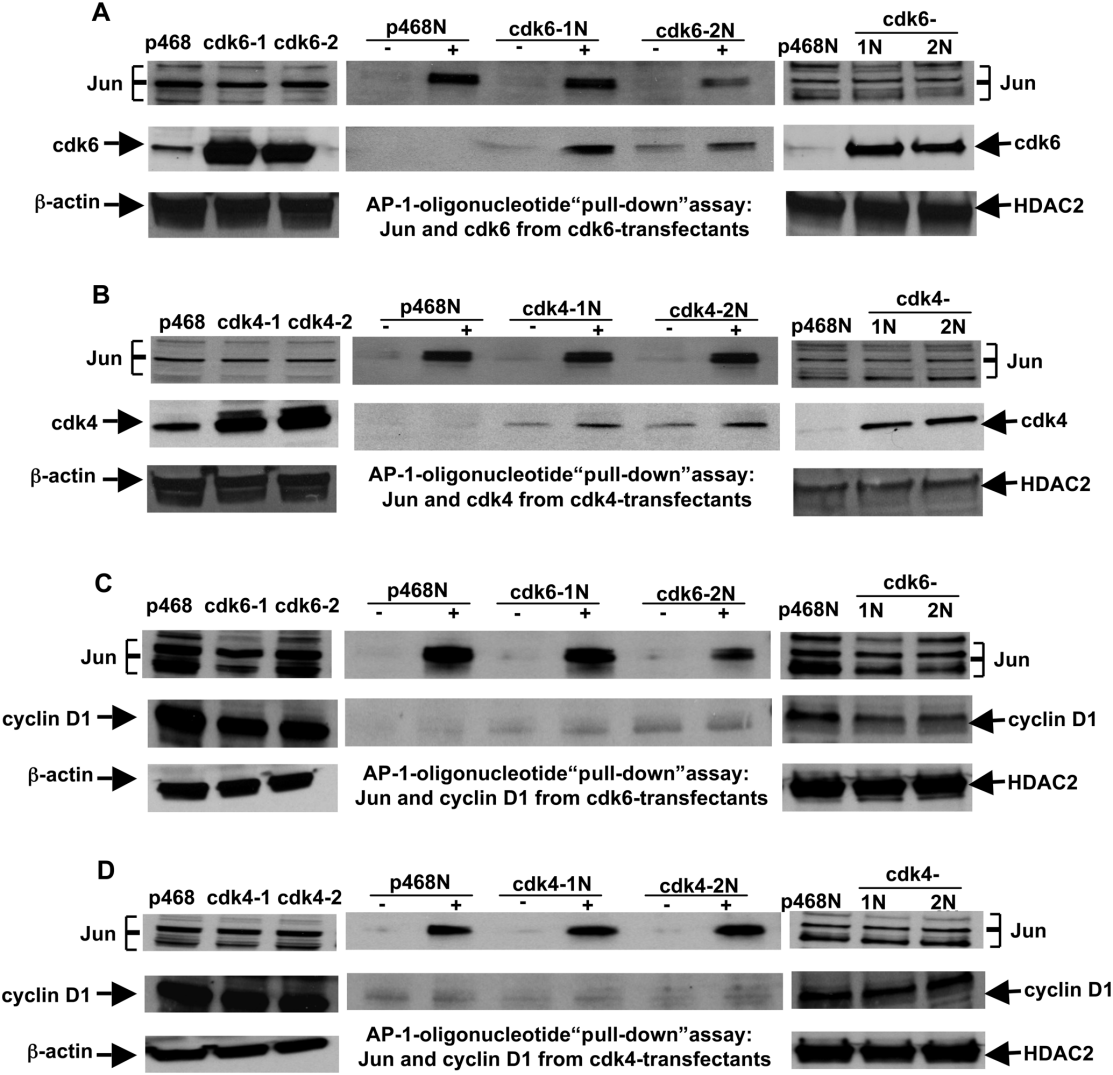
Association of cdk6 and cdk4, but not cyclin D1, with the AP-1 promoter sequence of the 17β-HSD2 gene. A biotin-labeled oligonucleotide representing the sequence was used in oligonucleotide “pull-down” assays with nuclear extracts from MDA-MB-468 parental cells and from 2 cell lines stably-transfected with cdk6 (panels (**A**) and (**C**)) and 2 cell lines transfected with cdk4 (panels (**B**) and (**D**)). The oligonucleotide and associated nuclear proteins were collected by binding to streptavidin-conjugated beads. The proteins associated with the oligonucleotide (shown in the lanes marked “+” in the 2 center immunoblots in each panel ((**A**) through (**D**)) were probed for Jun (top immunoblot in (**A**) through (**D**)) and either cdk6 (**A**), cdk4 (**B**), or cyclin D1 ((**C**) and (**D**)), respectively (bottom immunoblots). Lanes designated as “−” are negative control samples in which the nuclear extracts were incubated without the oligonucleotide but then subjected to collection using streptavidin-conjugated beads. Immunoblots to the left of the center blots show the levels of Jun, β-actin, and either (**A**) cdk6, (**B**) cdk4, or (**C**) and (**D**) cyclin D1 in whole cell extracts from the parental MDA-MB-468 cells (p468) and the cdk6 (cdk6-1 and cdk6-2) and cdk4 (cdk4-1 and cdk4-2) transfectants. Immunoblots to the right of the center blots show the levels of Jun, HDAC2, and either (**A**) cdk6, (**B**) cdk4, or (**C**) and (**D**) cyclin D1 in nuclear extracts from the parental MDA-MB-468 cells (p468N) and from the cdk6 (cdk6-1N and cdk6-2N) and cdk4 (cdk4-1N and cdk4-2N) transfectants.

To delineate the mechanism by which cdks affect SME gene transcript levels, experiments were performed to determine if cdks associated with the promoter region of an affected gene. The 17β-HSD2 promoter contains an activator protein (AP)-1 site involved in gene transcription [33]. A biotin-labeled oligonucleotide encompassing this sequence was synthesized and used in “pull-down” experiments with nuclear extracts from MDA-MB-468 parental and 2 cdk6- and 2 cdk4-transfectant cell lines. Both Jun, a component of AP-1, and cdk6 were found to associate with the 17β-HSD2 sequence (Figure 6A), suggesting that cdk6 may act to regulate transcripts through an association with the promoter sequence, either directly or through association with other proteins. That cdk6 is associated with the gene sequence through an interaction with Jun was not supported by coimmunoprecipitation experiments (data not shown). A similar analysis was performed using cdk4-overexpressing lines. The results in Figure 6B indicate an association of cdk4 with the 17β-HSD2 promoter sequence. The 17β-HSD2 sequence with associated nuclear proteins from MDA-MB-468 cells and from the cdk6 and cdk4 transfectant cell lines was also probed for cyclin D1 (Figures 6C and 6D, respectively). Cyclin D1 levels were not above background levels as seen in control samples, supporting the notion that the effects of cdk6 and cdk4 on gene expression in cdk6 and cdk4 overexpressing cells were independent of cyclin D1.

## Discussion

**Figure 7:**
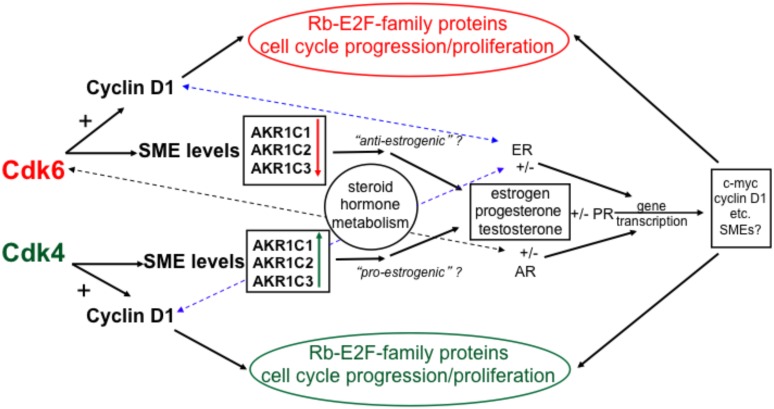
Regulatory functions of cdk6 and cdk4 in breast tumor epithelial cells: interactions of the cell cycle and steroid hormone metabolism and function. Cdk4 and cdk6 interact with cyclin D1 to regulate cell cycle progression and proliferation, through Rb and E2F-family proteins. The cdks can also regulate levels of SMEs, including the AKR1C-family of enzymes. Induction or suppression of AKR1C-family enzymes by cdk4 or cdk6, respectively, could induce either a pro- or anti-estrognic state in a breast tumor. The dashed blue lines indicate that the ER can interact with cyclin D1 to affect gene transcription, even in the absence of estrogen [40], [41]. The dashed black line indicates the interaction of the androgen receptor with cdk6, which can stimulate androgen receptor-directed gene transcription, independently of cyclin D1 [42]. Steroid hormones bound to cognate steroid receptors regulate transcription of many genes that can affect tumor cell growth and function, including those encoding proteins that directly affect the cell cycle, such as c-myc and cyclin D1 [38], [39] and perhaps SME genes themselves [54].

The G1-phase cdks (cdk6 and cdk4) had differential effects on the levels of transcripts encoding SMEs that are involved in estrogen metabolism, identifying a novel mechanism for pre-receptor control of steroid hormone action. Links between the cell cycle and steroid hormone action have been observed previously. Estrogen receptor (ER) ligation induces the activation of many genes that play roles in proliferation, including c-myc and cyclin D1 [38], [39]. In turn, cyclin D1 can alter ER-mediated transcription through interactions with ER co-regulatory and other transcriptional control proteins, even permitting transactivation of ER-regulated genes in the absence of estrogen [40], [41]. Cdk6 can associate with the androgen receptor, resulting in transactivation of responsive genes, independently of cdk6 binding to cyclin D1 [42]. Cyclin D1 and cdk6 can also modulate transcription through interaction with other transcription factors, including STAT3 [43] and Runx1 [44], respectively. Integration of the findings presented here with results of previous studies is shown in Figure 7, which illustrates that cdk6 and cdk4, associated with cyclin D1, regulate cell cycle progression, and also, perhaps independently of cyclin D1, modulate expression of genes encoding SMEs.

The precursor to steroid hormones is cholesterol, which is transported into the mitochondria and introduced into the metabolic pathways under study by CYP11A1, which converts cholesterol to pregnenolone, the first step in steroidogenesis [45]. A possible link between cholesterol and the cell cycle was suggested by Cirera-Salinas et al. [46], who showed that the microRNA miR-33, which regulates the expression of genes involved in fatty acid and cholesterol metabolism [47], also modulates expression of the genes encoding cdk6 and cyclin D1. The results described here suggest that this link may extend to other steps beyond the biogenesis of cholesterol.

The growth and function of steroid hormone-responsive tissues and tumors are dependent on steroid hormone levels, which are determined by numerous enzymes involved in steroid metabolism. Regulating levels of key SMEs provides pre-receptor mechanisms for control of steroid hormone action [21], [22]. In the mammary gland, the abundant AKR1C3 isoform can reduce estrone to active 17β-estradiol and catalyze the reaction of Δ^4^-androstene-3,17-dione to testosterone, which can then be aromatized to 17β-estradiol by CYP19 aromatase. AKR1C3, along with AKR1C1, also reduces progesterone to the less active 20α-hydroxyprogesterone form. Changes in the levels of these enzymes can thus alter hormone ratios to a pro-estrogenic state favoring proliferation [23], [24]. Type 1 and type 2 17β-HSD, which catalyze the reduction of estrone to active estradiol and the reverse reaction, respectively, also play a role in determining hormone balance in the mammary gland [28], as do several CYP enzymes, including CYP1B1, an estrogen hydroxylase found in breast tissue [26]. Many of these enzymes and/or their transcripts are altered in some fraction of breast tumors [26]–[28].

The almost complete repression of AKR1C1 and AKR1C3 transcripts by cdk6 in MDA-MB-468 cells could have a major impact in lowering the estrogen to progesterone ratio. Conversely, dramatic increases seen after cdk4 overexpression would favor a pro-estrogenic state. These observations are consistent with the notion that increased cdk4 but *decreased* cdk6 levels are associated with the tumorigenic phenotype of breast cancer cells [3], [12]. As noted above, cdk6 protein levels were reduced in most breast tumor clinical samples examined, especially in the cell nuclei, as compared to cells in normal breast tissue, regardless of tumor cell morphology [12]. In contrast, An et al. [48] described frequent amplification of the cdk4 gene and high cdk4 expression in breast cancers, especially in tumors of higher histological grade. Byrnes et al. [49] showed that overexpression of AKR1C3 in MCF-7 cells resulted in a pro-estrogenic state with increased cell proliferation in response to estrone or 17β-estradiol. Zhang et al. [50] demonstrated that changing the balance of 17β-HSD1 and 17β-HSD2 isoforms in breast cancer cell lines altered the ratio of estradiol to estrone produced by the cells. Changes in steroid metabolism in the cdk-transfected breast tumor cells described here will be presented in a subsequent publication. Since overexpression of cdk6 or cdk4 induces concomitant changes in several SMEs, a mass spectrometric approach to delineating the final changes in steroid hormone production will be utilized.

Analysis of SME gene expression was performed in 3 tumor-derived cell lines, with similar results. It was noteworthy that the effects of altered cdk4 expression in normal mammary epithelial cells was similar to that seen in the tumor cell lines with respect to AKR1C-family proteins but differed with respect to others SMEs. These differences may reflect other oncogenic events that have occurred in the tumor cell lines. Of importance, the results establish that cdk-mediated regulation of SMEs is an event that can occur in normal cells. Altered expression of cyclin D1, which is observed in many breast tumors [2], had little effect on most SME transcript levels and cyclin D1 was not associated with the Jun/cdk promoter complexes in oligonucleotide “pull-down” experiments. Lim et al. [42] reported that cdk-enhanced transcriptional activity of the androgen receptor in prostate cells was independent of its kinase activity. Inhibitors designed to target cdk enzymatic activities [51] will not be useful in therapy if cdk function is independent of kinase activity.

Changes in cdks may thwart standard treatments designed to alter hormone levels by inhibiting SMEs. Furthermore, because of the functional plasticity of some SMEs, changes in their levels may affect other aspects of tumor activity. For example, AKR1C3 is also a prostaglandin F synthase and is involved in prostaglandin alterations that stimulate tumor growth, angiogenesis, and invasiveness [23], [49], [52]. AKR1C-family and certain CYP enzymes, including CYP1B1, are involved in the activation of procarcinogens and the degradation of anticancer drugs [22], [25], [26], [53]. From estradiol, CYP1B1 catalyzes the formation of genotoxic catecholestrogens which can lead to DNA and protein damage [26]. Thus, alterations in cell cycle proteins may, through effects on these SME functions, have an impact on both tumorigenesis and treatment efficacy. Understanding the mechanisms involved in cdk regulation of SME transcript levels may suggest new treatments that combine strategies to inhibit both pre- and post-receptor mechanisms of steroid hormone action.

## Supporting Information

Figure S1 The pattern of SME gene transcripts in breast cancer cells, assayed at 2 times after transfection, is altered in stably-transfected cell lines overexpressing cdk6 protein. MDA-MB-468 breast epithelial cells were stably transfected with a sequence encoding cdk6. **(A)** AKR1C1, AKR1C2, and AKR1C3 transcript levels in duplicate cultures of parental MDA-MB-468 cells (468-1 and 468-2) and of 2 stably-transfected cdk6-overexpressing cell lines harvested at 2 different times, separated by several weeks of growth (468-cdk6-1a and 468-cdk6-1b and 468-cdk6-4a and 468-cdk6-4b), were detected and quantitated by qRT-PCR. **(B)** CYP19 and 17β-HSD2 transcript levels were quantitated in the 6 cultures. The data is expressed as the mean ± SEM, n = 3 times/group; *p<0.05 and **p<0.01.
(TIF)

Figure S2 Efficiency of transfection of (A) MDA-MB-468 cells and (B) normal human mammary epithelial cells. The efficiencies of transfection were monitored by flow cytometry after transfection with a plasmid encoding green fluorescent protein (GFP). **(A)** For MDA-MB-468 cells, comparison of mock-transfected (left panel) and pGFP-transfected (right panel) cells indicated that 45% of the cells were transfected, as determined at 2 days after transfection. **(B)** For HMECs, comparison of mock-transfected (left panel) and pGFP-transfected (right panel) cells indicated that 47.9% of the cells were transfected, as determined at 2 days after transfection.
(TIF)

Figure S3 Enhanced proliferation of MDA-MB-468 cells stably transfected to overexpress cdk4. Stock cultures of parental MDA-MB-468 cells and the 3 indicated cdk4 stably transfected cell lines were thawed and grown for 1 week in growth medium. They were then seeded at 0.2×10^5^ cells/well in 6-well tissue culture plates. At the times indicated on the graph, cells were removed by trypsin treatment and cell numbers were determined using a hemocytometer. Initial cell numbers for each culture were normalized to 100.
(TIF)

Figure S4 Immunoblot analysis of cdk4 and cdk6 levels in parental MDA-MB-468 cells and in cdk6- and cdk4-transfected cells. Growing cultures of parental MDA-MB-468 cells (p468) and of cdk6- and cdk4-transfected cells were harvested and extracts were assessed for cdk4, cdk6, and β-actin levels, as described in the Materials and Methods. The results indicate little substantial change in cdk6 levels in cdk4-transfected cells or in cdk4 levels in cdk6-transfected cells.
(TIF)

Figure S5 SME gene expression in MDA-MB-468 cells stably-transfected with wild-type (WT) or dominant-negative (DN) forms of cdk6. Parental MDA-MB-468 cells (p468) were stably transfected with either the WT or DN form of cdk6 and SME gene expression was evaluated by RT-PCR for the AKR1C1, AKR1C2, AKRIC3, and β-actin genes (panel **(A)**) and for the 17β-HSD1, 17β-HSD2, and β-actin genes (panel **(B)**), as described in the Materials and Methods.
(TIF)

Table S1 Primers used for RT-PCR Analyses.
(TIF)

Table S2 qRT-PCR primers and probe for AKR1C2.
(TIF)

Table S3 A. Antibodies used for immunoblot analyses. B. Antibodies used for immunohistochemical analyses.
(TIF)

Table S4 Differential SME transcript levels in parental MDA-MB-468 breast tumor cells and cells overexpressing cdk6 or cdk4, as determined by qRT-PCR.
(TIF)

Table S5 Differential SME transcript levels in 3 breast tumor-derived cell lines and in normal human mammary epithelial cells (HMECs), as determined by qRT-PCR.
(TIF)

File S1 Supplementary Materials and Methods.
(DOC)

## References

[1] Ho A, Dowdy SF (2002) Regulation of (G)1 cell-cycle progression by oncogenes and tumor suppressor genes. Curr Opin Genet Dev 12: 47–52. 11790554 10.1016/s0959-437x(01)00263-5

[2] Fu M, Wang C, Li Z, Sakamaki T, Pestell RG (2004) Cyclin D1: normal and abnormal functions. Endocrinol 145: 5439–5447. 10.1210/en.2004-0959 15331580

[3] Yu Q, Sicinska E, Geng Y, Ahnstrom M, Zagozdzon A, et al (2006) Requirement for cdk4 kinase function in breast cancer. Cancer Cell 9: 23–32. 16413469 10.1016/j.ccr.2005.12.012

[4] Grossel MJ, Hinds PW (2006) Beyond the cell cycle: A new role for cdk6 in differentiation. J Cell Biochem 97: 485–493. 16294322 10.1002/jcb.20712

[5] Anders L, Ke N, Hydbring P, Choi YJ, Widlund HR, et al (2011) A systematic screen for CDK4/6 substrates links FOXM1 phosphorylation to senescence suppression in cancer cells. Cancer Cell 20: 620–634. 22094256 10.1016/j.ccr.2011.10.001 PMC3237683

[6] Malumbres M, Barbacid M (2005) Mammalian cyclin-dependent kinases. Trends Biochem Sci 30: 630–641. 16236519 10.1016/j.tibs.2005.09.005

[7] Matushansky I, Radparvar F, Skoultci AI (2000) Manipulating the onset of cell cycle withdrawal in differentiated erythroid cells with cyclin-dependent kinases and inhibitors. Blood 96: 2755–2764. 11023509

[8] Ericson KK, Krull D, Slomiany P, Grossel MJ (2003) Expression of cyclin-dependent kinase 6, but not cyclin-dependent kinase 4, alters morphology of cultured mouse astrocytes. Mol Cancer Res 1: 654–664. 12861051

[9] Quelle DE, Ashmun RA, Shurtleff SA, Kato JY, Bar-Sagi D, et al (1993) Overexpression of mouse D-type cyclins accelerates G1 phase in rodent fibroblasts. Genes Dev 7: 1559–1571. 8339933 10.1101/gad.7.8.1559

[10] Grossel MJ, Baker GL, Hinds PW (1999) Cdk6 can shorten G1 phase dependent upon the N-terminal INK4 interaction domain. J Biol Chem 274: 29960–29967. 10514479 10.1074/jbc.274.42.29960

[11] Nagasawa M, Gelfand EW, Lucas JJ (2001) Accumulation of high levels of the p53 and p130 growth-suppressing proteins in cell lines stably over-expressing cyclin-dependent kinase 6 (cdk6). Oncogene 20: 2889–2899. 11420701 10.1038/sj.onc.1204396

[12] Lucas JJ, Domenico J, Gelfand EW (2004) Cyclin-dependent kinase 6 inhibits proliferation of human mammary epithelial cells. Mol Cancer Res 2: 105–114. 14985467

[13] Chilosi M, Doglioni C, Yan Z, Lestani M, Menestrina F, et al (1998) Differential expression of cyclin-dependent kinase 6 in cortical thymocytes and T-cell lymphoblastic lymphoma/leukemia. Am J Pathol 152: 209–217. 9422538 PMC1858129

[14] Timmermann S, Hinds PW, Munger K (1997) Elevated activity of cyclin-dependent kinase 6 in human squamous cell carcinoma lines. Cell Growth Differ 8: 361–370. 9101082

[15] Easton J, Wei T, Lahti JM, Kidd VJ (1998) Disruption of the cyclin D/cyclin-dependent kinase/INK4/retinoblastoma protein regulatory pathway in human neuroblastoma. Cancer Res 58: 2624–2632. 9635589

[16] Grigoriadis A, Mackay A, Reis-Filho JS, Steele D, Iseli C, et al (2006) Establishment of the epithelial-specific transcriptome of normal and malignant breast cells based on MPSS and array expression data. Breast Cancer Res 8: R56. 17014703 10.1186/bcr1604 PMC1779497

[17] Tay SP, Yeo CW, Chai C, Chua PJ, Tan HM, et al.. (2010) Parkin enhances the expression of cyclin-dependent kinase 6 and negatively regulates the proliferation of breast cancer cells. J Biol Chem 285, 29231–29238. 10.1074/jbc.M110.108241 PMC2937954 20630868

[18] Tomita T (2004) Cyclin-dependent kinase (cdk6) and p16 in pancreatic endocrine neoplasms. Pathology 36: 566–570. 15841692 10.1080/00313020400011342

[19] Wang X, Sistrunk C, Rodriguez-Puebla ML (2011) Unexpected reduction of skin tumorigenesis on expression of cyclin-dependent kinase 6 in mouse epidermis. Am J Pathol 178: 345–354. 21224071 10.1016/j.ajpath.2010.11.032 PMC3069882

[20] Ito K, Maruyama Z, Sakai A, Izumi S, Moriishi T, et al. (2013) Overexpression of *Ckd6* and *Ccnd1* in chondrocytes inhibited chondrocyte maturation and caused p53-dependent apoptosis without enhancing proliferation. Oncogene (In press). 10.1038/onc.2013.130 23624920

[21] Sasano H, Suzuki T, Nakata T, Moriya T (2006) New developments in intracrinology of breast carcinoma. Breast Cancer 13: 129–136. 16755106 10.2325/jbcs.13.129

[22] Penning TM, Drury JE (2007) Human aldo-keto reductases: Function, gene regulation, and single nucleotide polymorphisms. Arch Biochem Biophys 464: 241–250. 17537398 10.1016/j.abb.2007.04.024 PMC2025677

[23] Byrns MC, Penning TM (2009) Type 5 17β-hydroxysteroid dehydrogenase/prostaglandin F synthase (AKR1C3): Role in breast cancer and inhibition by non-steroidal anti-inflammatory drug analogs. Chem Biol Interact 178: 221–227. 19010312 10.1016/j.cbi.2008.10.024 PMC3076957

[24] Penning TM, Byrns MC (2009) Steroid hormone transforming aldo-keto reductases and cancer. Ann NY Acad Sci 1155: 33–42. 19250190 10.1111/j.1749-6632.2009.03700.x PMC3038333

[25] Shimada T, Hayes CL, Yamazaki H, Amin S, Hecht SS, et al (1996) Activation of chemically diverse procarcinogens by human cytochrome P-450 1B1. Cancer Res 56: 2979–2984. 8674051

[26] Sissung TM, Price DK, Sparreboom A, Figg WD (2006) Pharmacogenetics and regulation of human cytochrome P450 1B1: implications in hormone-mediated tumor metabolism and a novel target for therapeutic intervention. Mol Cancer Res 4: 135–150. 16547151 10.1158/1541-7786.MCR-05-0101

[27] Lin HK, Steckelbroeck S, Fung KM, Jones AN, Penning TM (2004) Characterization of a monoclonal antibody for human aldo-keto reductase AKR1C3 (type 2 3alpha-hydroxysteroid dehydrogenase/type 5 17beta-hydroxysteroid dehydrogenase); immunohistochemical detection in breast and prostate. Steroids 69: 795–801. 15582534 10.1016/j.steroids.2004.09.014

[28] Jannson A (2009) 17Beta-hydroxysteroid dehydrogenase enzymes and breast cancer. J Steroid Biochem Mol Biol 114: 64–67. 19167496 10.1016/j.jsbmb.2008.12.012

[29] Li G, Domenico J, Jia Y, Lucas JJ, Gelfand EW (2009) NF-kappaB-dependent induction of cathelicidin-related antimicrobial peptide in murine mast cells by lipopolysaccharide. Int Arch Allergy Immunol 150: 122–132. 19439978 10.1159/000218115 PMC2814151

[30] Li G, Lucas JJ, Gelfand EW (2005) Protein kinase Cα, βI, and βII isozymes regulate cytokine production in mast cells through MEKK2/ERK5-dependent and -independent pathways. Cell Immunol 238: 10–18. 16430878 10.1016/j.cellimm.2005.12.001

[31] Bauman DR, Rudnick SI, Szewczuk LM, Jin Y, Gopishetty S, et al (2005) Development of nonsteroidal anti-inflammatory drug analogs and steroid carboxylates selective for human aldo-keto reductase isoforms: potential anti-neoplastic agents that work independently of cyclooxygenase isozymes. Mol Pharmacol 67: 60–68. 15475569 10.1124/mol.104.006569

[32] Stoffel-Wagner B, Watzka M, Steckelbroeck S, Ludwig M, Clusmann H, et al (2003) Allopregnanolone serum levels and expression of 5α-reductase and 3α-hydroxysteroid dehydrogenase isoforms in hippocampal and temporal cortex of patients with epilepsy. Epilepsy Res 54: 11–19. 12742591 10.1016/s0920-1211(03)00036-6

[33] Yang S, Fang Z, Gurates B, Tamura M, Miller J, et al (2001) Stromal PRs mediate induction of 17β-hydroxysteroid dehydrogenase type 2 expression in human endometrial epithelium: a paracrine mechanism for inactivation of E2. Mol Endocrinol15: 2093–2105. 10.1210/mend.15.12.0742 11731611

[34] Penning TM, Burczynski M, Jez JM, Hung CF, Lin HK, et al (2000) Human 3alpha-hydroxysteroid dehydrogenase isoforms (AKR1C1–AKR1C4) of the aldo-keto reductase superfamily: functional plasticity and tissue distribution reveals roles in the inactivation and formation of male and female sex hormones. Biochem J 351: 67–77. 10998348 10.1042/0264-6021:3510067 PMC1221336

[35] Lim S, Kaldis P (2013) Cdks, cyclins and CKIs: roles beyond cell cycle regulation. Development 140: 3079–3093. 23861057 10.1242/dev.091744

[36] Kasten M, Giordano A (2001) Cdk10, a Cdc2-related kinase, associates with the Ets2 transcription factor and modulates its activity. Oncogene 20: 1832–1838. 11313931 10.1038/sj.onc.1204295

[37] Bagella L, Giacinti C, Simone C, Giordano A (2006) Identification of murine cdk10:association with Ets2 transcription factor and effects on the cell cycle. J Cell Biochem 99: 978–985. 16741970 10.1002/jcb.20981

[38] Dubik D, Shiu RP (1992) Mechanism of estrogen activation of c-myc oncogene expression. Oncogene 7: 1587–1594. 1630819

[39] Altucci L, Addeo R, Cicatiello L, Dauvois S, Parker MG, et al (1996) 17-beta Estradiol induces cyclin D1 gene transcription, p36D1-p34cdk4 complex activation and p105Rb phosphorylation during mitogenic stimulation of G(1)-arrested human breast cancer cells. Oncogene 12: 2315–2324. 8649771

[40] Neuman E, Ladha MH, Lin N, Upton TM, Miller SJ, et al (1997) Cyclin D1 stimulation of estrogen receptor transcriptional activity independent of cdk4. Mol Cell Biol 17: 5338–5347. 9271411 10.1128/mcb.17.9.5338 PMC232384

[41] Zwijsen RM, Buckle RS, Hijmans EM, Loomans CJ, Bernards R (1998) Ligand-independent recruitment of steroid receptor coactivators to estrogen receptor by cyclin D1. Genes Dev 12: 3488–3498. 9832502 10.1101/gad.12.22.3488 PMC317237

[42] Lim JT, Manskhani M, Weinstein IB (2005) Cyclin-dependent kinase 6 associates with the androgen receptor and enhances its transcriptional activity in prostate cancer cells. Proc Natl Acad Sci USA 102: 5156–5161. 15790678 10.1073/pnas.0501203102 PMC556011

[43] Bienvenu F, Gascan H, Coqueret O (2001) Cyclin D1 represses STAT3 activation through a cdk4-independent mechanism. J Biol Chem 276: 16840–16847. 11279133 10.1074/jbc.M100795200

[44] Fujimoto T, Anderson K, Jacobsen SE, Nishikawa S, Nerlov C (2007) Cdk6 blocks myeloid differentiation by interfering with Runx1 DNA binding and Runx1-C/EBPα interaction. EMBO J 26: 2361–2370. 17431401 10.1038/sj.emboj.7601675 PMC1864973

[45] Miller WL, Auchus RJ (2011) The molecular biology, biochemistry, and physiology of human steroidogenesis and its disorders. Endocr Rev 32: 81–151. 21051590 10.1210/er.2010-0013 PMC3365799

[46] Cirera-Salinas D, Pauta M, Allen RM, Salerno AG, Ramirez CM, et al (2012) Mir-33 regulates cell proliferation and cell cycle progression. Cell Cycle 11: 922–933. 22333591 10.4161/cc.11.5.19421 PMC3323796

[47] Rotllan N, Fernandez-Hernando C (2012) MicroRNA regulation of cholesterol metabolism. Cholesterol 2012: 847849. 22919472 10.1155/2012/847849 PMC3420088

[48] An HX, Beckmann MW, Reifenberger G, Bender HG, Niederacher D (1999) Gene amplification and overexpression of CDK4 in sporadic breast carcinomas is associated with high tumor cell proliferation. Am J Pathol 154: 113–118. 9916925 10.1016/S0002-9440(10)65257-1 PMC1868599

[49] Byrns MC, Duan L, Lee SH, Blair IA, Penning TM (2010) Aldo-keto reductase 1C3 expression in MCF-7 cells reveals roles in steroid hormone and prostaglandin metabolism that may explain its over-expression in breast cancer. J Steroid Biochem Mol Biol 118: 177–187. 20036328 10.1016/j.jsbmb.2009.12.009 PMC2819162

[50] Zhang CY, Chen J, Yin DC, Lin SX (2012) The contribution of 17beta-hydroxysteroid dehydrogenase type 1 to the estradiol-estrone ration in estrogen-sensitive breast cancer cells. 7: e29835. 10.1371/journal.pone.0029835 PMC3253791 22253796

[51] McInnes C (2008) Progress in the evaluation of CDK inhibitors as anti-tumor agents. Drug Discov Today 13: 875–881. 18639646 10.1016/j.drudis.2008.06.012

[52] Milne SA, Jabbour HN (2003) Prostaglandin (PG) F_2α_ receptor expression and signaling in human endometrium: role of PGF_2α_ in epithelial cell proliferation. J Clin Endocrinol Metab 88: 1825–1832. 12679480 10.1210/jc.2002-021368

[53] Novotna R, Wsol V, Xiong G, Maser E (2008) Inactivation of the anticancer drugs doxorubicin and oracin by aldo-keto reductase (AKR) 1C3. Toxicol Lett 18: 1–6. 10.1016/j.toxlet.2008.06.858 18616992

[54] Kang KW, Kim YG (2008) Bioequivalence studies of tibolone in premenopausal women and effects on expression of the tibolone-metabolizing enzyme AKR1C (aldo-keto reductase) family caused by estradiol. J Clin Pharmacol 48: 1430–1437. 18832293 10.1177/0091270008323262

