# Supplementary figures and images for: Modulated Expression of Genes Encoding Estrogen Metabolizing Enzymes by G1-Phase Cyclin-Dependent Kinases 6 and 4 in Human Breast Cancer Cells
Source: PLoS One. 2014 May 21;9(5):e97448. doi: 10.1371/journal.pone.0097448 (PMC4029737; doi:10.1371/journal.pone.0097448)

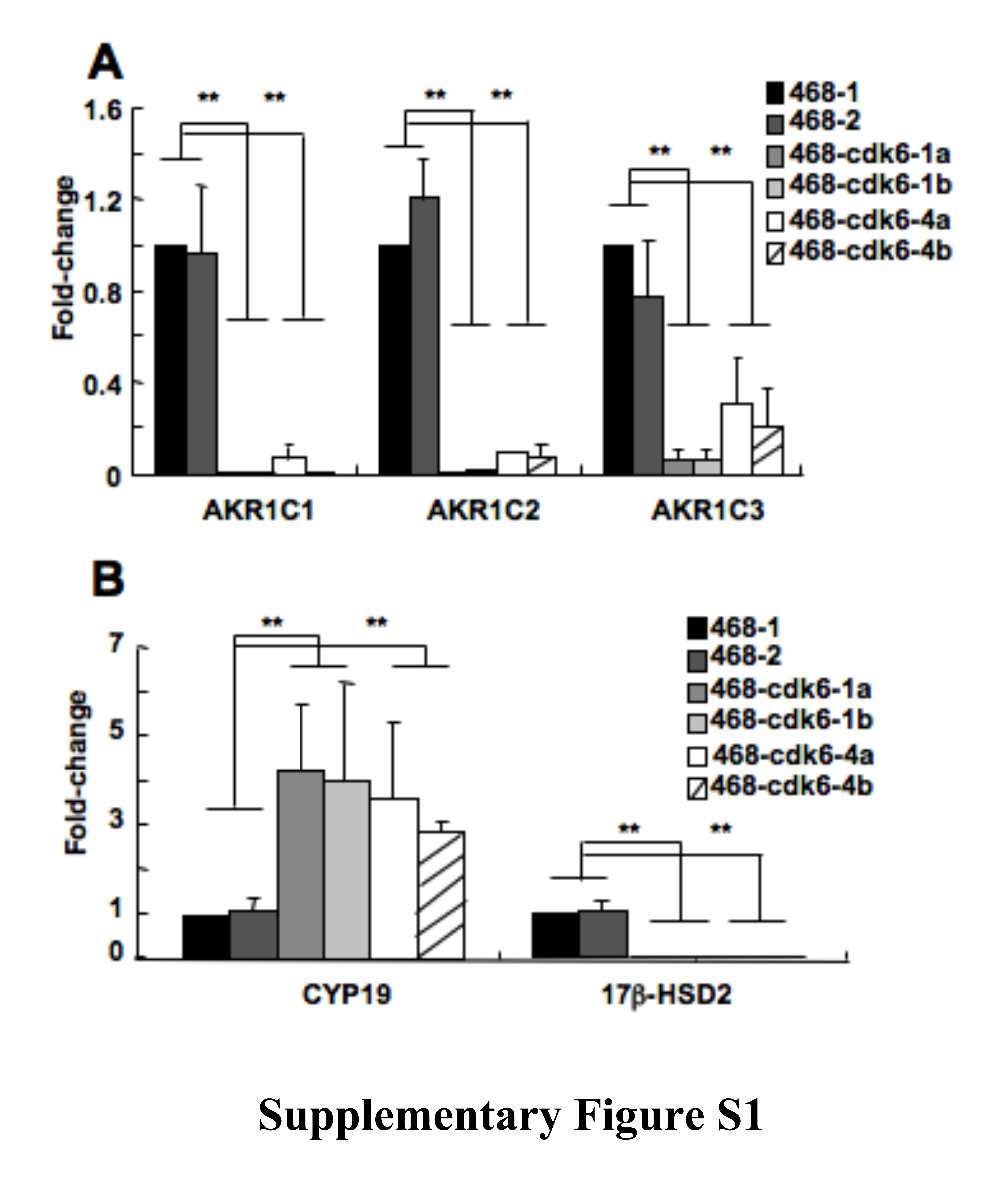

Supplement: Figure S1 — The pattern of SME gene transcripts in breast cancer cells, assayed at 2 times after transfection, is altered in stably-transfected cell lines overexpressing cdk6 protein. MDA-MB-468 breast epithelial cells were stably transfected with a sequence encoding cdk6. (A) AKR1C1, AKR1C2, and AKR1C3 transcript levels in duplicate cultures of parental MDA-MB-468 cells (468-1 and 468-2) and of 2 stably-transfected cdk6-overexpressing cell lines harvested at 2 different times, separated by several weeks of growth (468-cdk6-1a and 468-cdk6-1b and 468-cdk6-4a and 468-cdk6-4b), were detected and quantitated by qRT-PCR. (B) CYP19 and 17β-HSD2 transcript levels were quantitated in the 6 cultures. The data is expressed as the mean ± SEM, n = 3 times/group; *p<0.05 and **p<0.01. (TIF) [file pone.0097448.s001.tif]

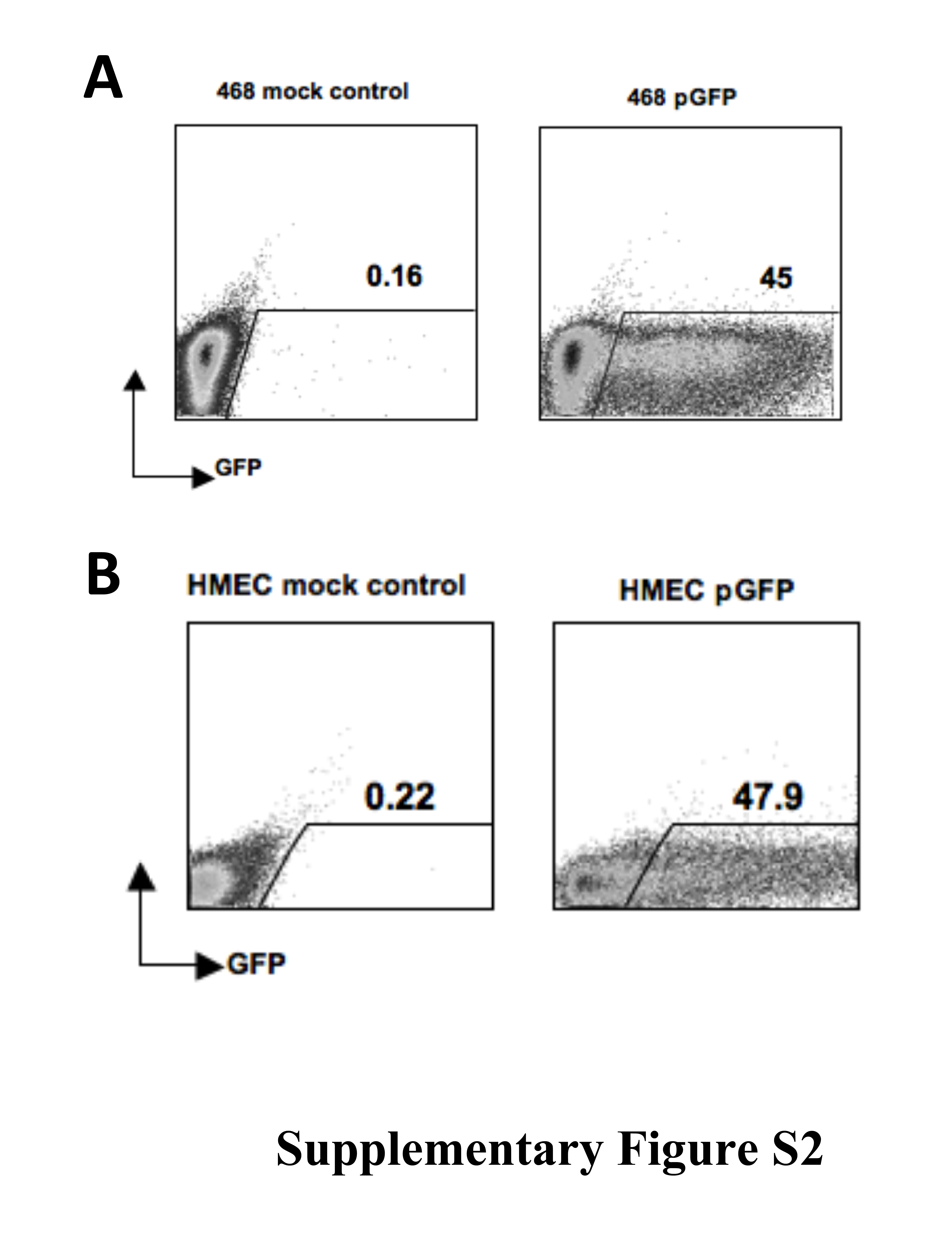

Supplement: Figure S2 — Efficiency of transfection of (A) MDA-MB-468 cells and (B) normal human mammary epithelial cells. The efficiencies of transfection were monitored by flow cytometry after transfection with a plasmid encoding green fluorescent protein (GFP). (A) For MDA-MB-468 cells, comparison of mock-transfected (left panel) and pGFP-transfected (right panel) cells indicated that 45% of the cells were transfected, as determined at 2 days after transfection. (B) For HMECs, comparison of mock-transfected (left panel) and pGFP-transfected (right panel) cells indicated that 47.9% of the cells were transfected, as determined at 2 days after transfection. (TIF) [file pone.0097448.s002.tif]

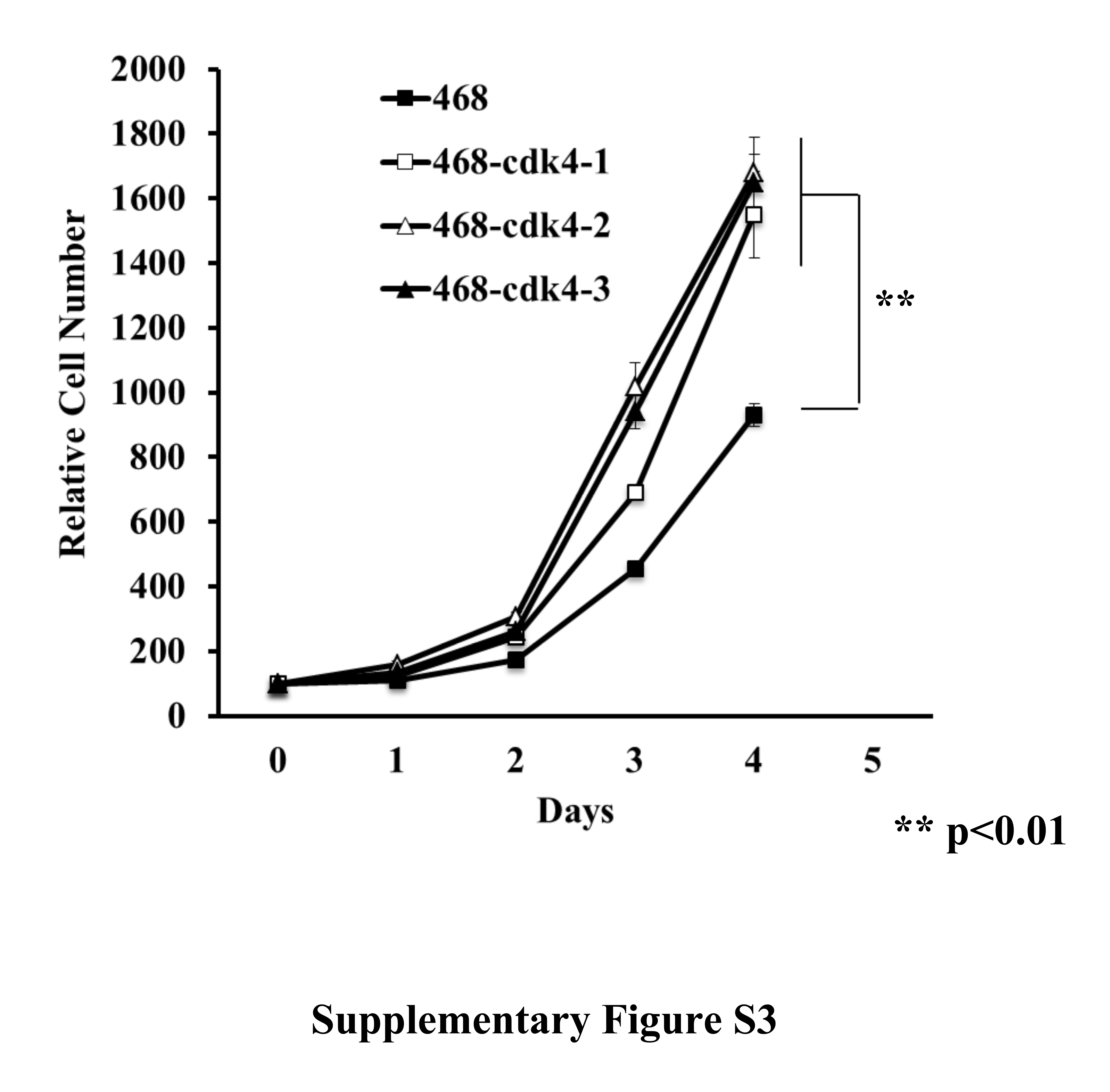

Supplement: Figure S3 — Enhanced proliferation of MDA-MB-468 cells stably transfected to overexpress cdk4. Stock cultures of parental MDA-MB-468 cells and the 3 indicated cdk4 stably transfected cell lines were thawed and grown for 1 week in growth medium. They were then seeded at 0.2×10^5^ cells/well in 6-well tissue culture plates. At the times indicated on the graph, cells were removed by trypsin treatment and cell numbers were determined using a hemocytometer. Initial cell numbers for each culture were normalized to 100. (TIF) [file pone.0097448.s003.tif]

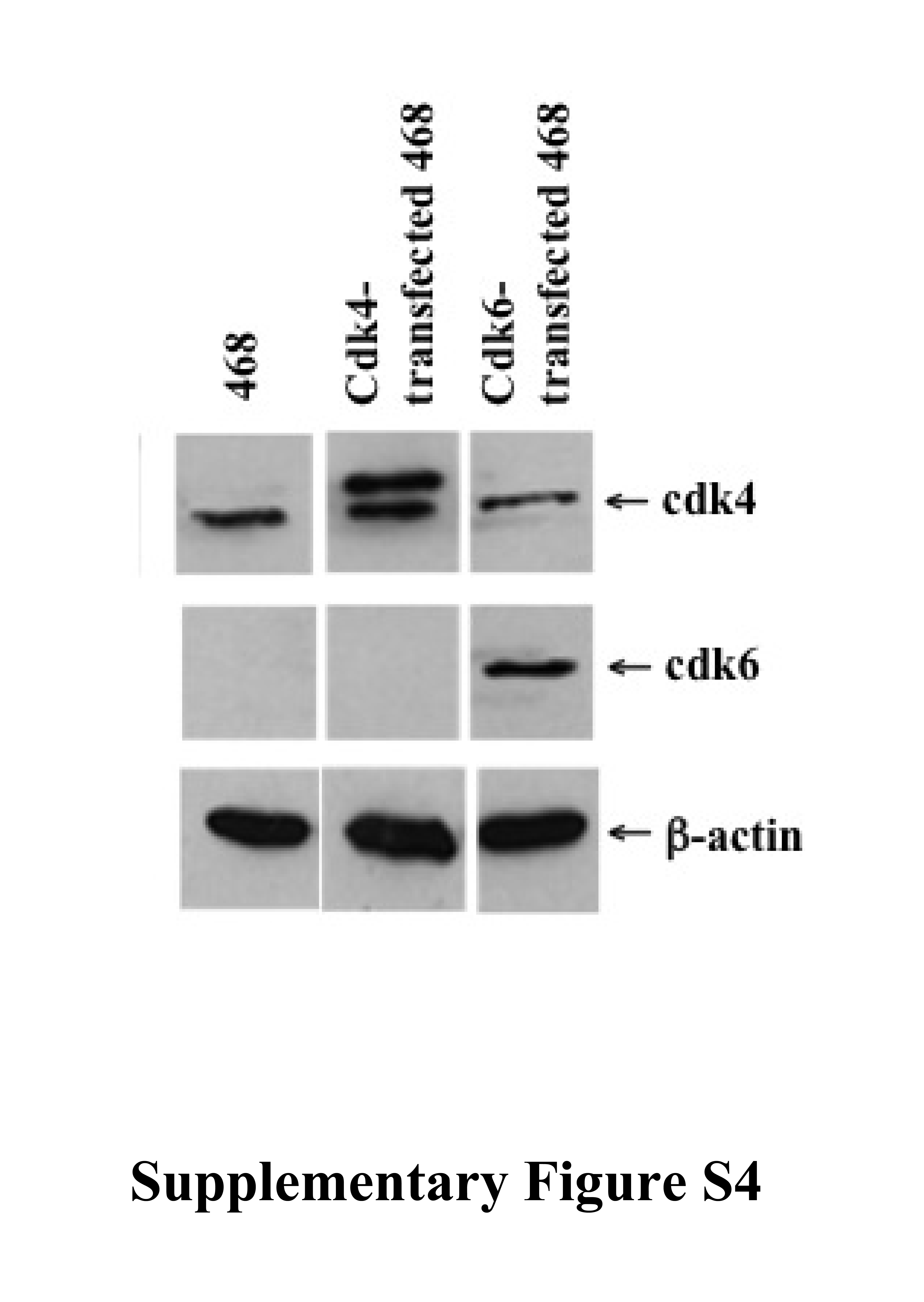

Supplement: Figure S4 — Immunoblot analysis of cdk4 and cdk6 levels in parental MDA-MB-468 cells and in cdk6- and cdk4-transfected cells. Growing cultures of parental MDA-MB-468 cells (p468) and of cdk6- and cdk4-transfected cells were harvested and extracts were assessed for cdk4, cdk6, and β-actin levels, as described in the Materials and Methods. The results indicate little substantial change in cdk6 levels in cdk4-transfected cells or in cdk4 levels in cdk6-transfected cells. (TIF) [file pone.0097448.s004.tif]

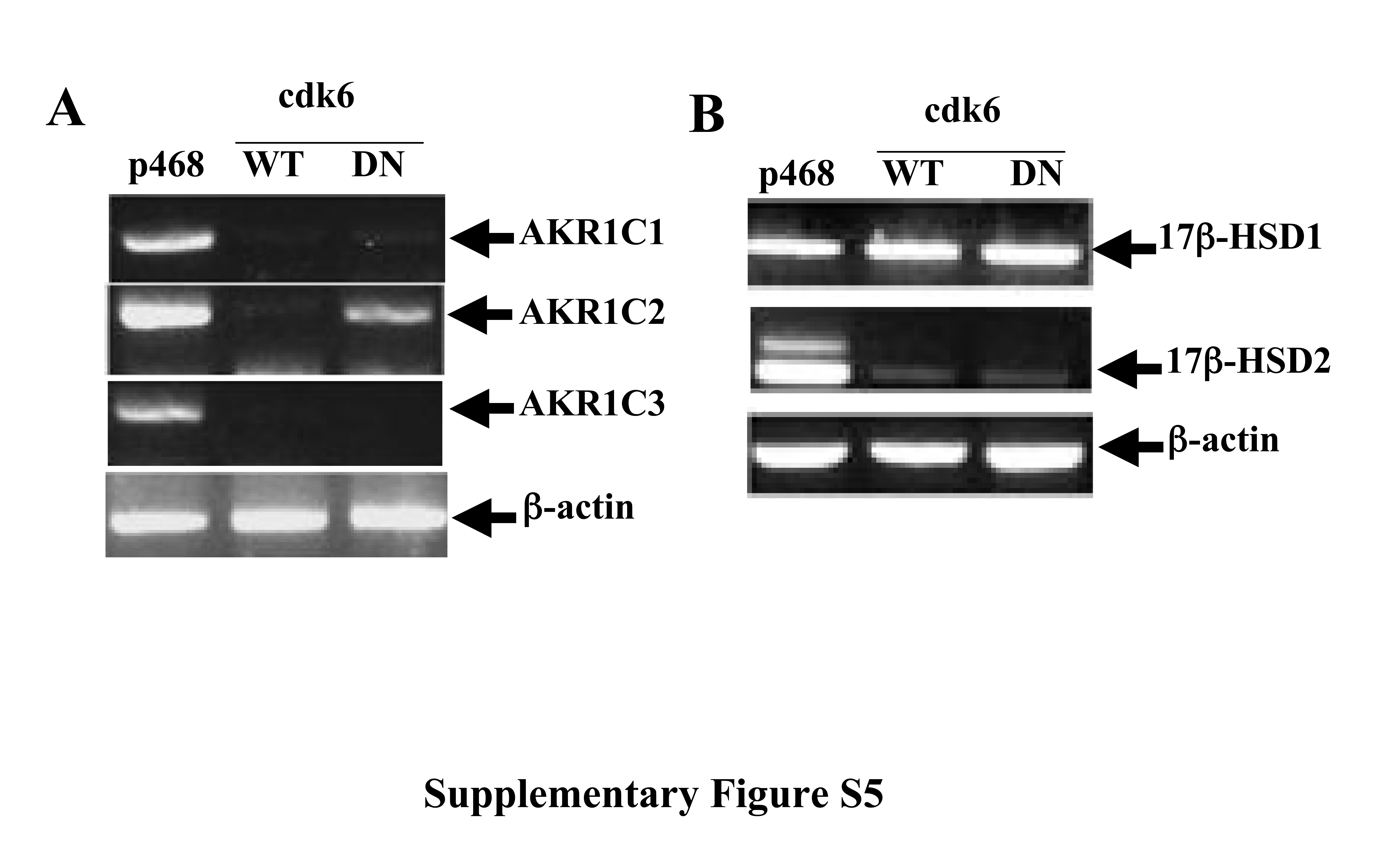

Supplement: Figure S5 — SME gene expression in MDA-MB-468 cells stably-transfected with wild-type (WT) or dominant-negative (DN) forms of cdk6. Parental MDA-MB-468 cells (p468) were stably transfected with either the WT or DN form of cdk6 and SME gene expression was evaluated by RT-PCR for the AKR1C1, AKR1C2, AKRIC3, and β-actin genes (panel (A)) and for the 17β-HSD1, 17β-HSD2, and β-actin genes (panel (B)), as described in the Materials and Methods. (TIF) [file pone.0097448.s005.tif]

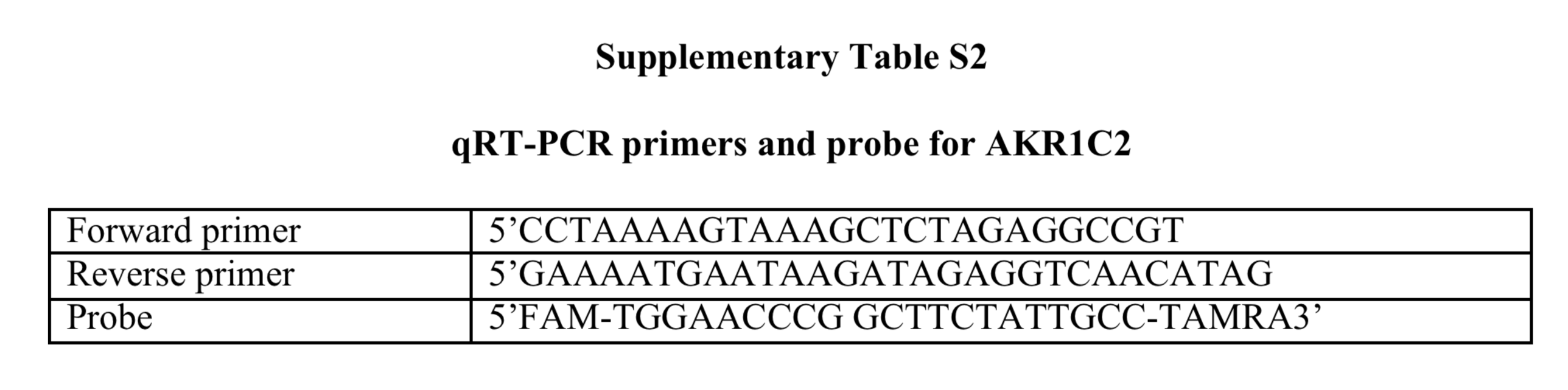

Supplement: Table S2 — qRT-PCR primers and probe for AKR1C2. (TIF) [file pone.0097448.s007.tif]
