# Supplementary material for: Modulated Expression of Genes Encoding Estrogen Metabolizing Enzymes by G1-Phase Cyclin-Dependent Kinases 6 and 4 in Human Breast Cancer Cells
Source: PLoS One. 2014 May 21;9(5):e97448. doi: 10.1371/journal.pone.0097448 (PMC4029737; doi:10.1371/journal.pone.0097448)
Supplement: File S1 — Supplementary Materials and Methods. (DOC) [file pone.0097448.s011.doc]

**Supplementary Information**

**Modulated Expression of Genes Encoding Estrogen Metabolizing Enzymes by G1-Phase Cyclin-Dependent Kinases 6 and 4 in Human Breast Cancer Cells**

Yi Jia*, Joanne Domenico, Christina Swasey, Meiqin Wang,

Erwin W. Gelfand and Joseph J. Lucas

Division of Cell Biology, Department of Pediatrics, National Jewish Health,

Denver, CO, 80206 USA

**Supplementary Materials and Methods**

***Cell Transfection***

Stably-transfected MDA-MB-468-derived cell lines were generated by electroporation using a Cell-Porator (Invitrogen) (1), or an Amaxa Nucleofector-II Device (Lonza/Amaxa) with Cell Line Nucleofector Solution V (#VCA-1003) and Program X-005. MDA-MB-453 cells were transfected using Solution C (#VCA-1004) and Program X-001 and MCF-7 cells using Solution V and Program P-020. After recovery for 1-3 days in complete medium, cells were seeded at low density in medium containing 300, 600, or 800 μg/ml G418, for MDA-MB-468, MDA-MB-453, or MCF-7 cells, respectively. These doses were effective for killing nontransfected cells [12]. Colonies were isolated and grown to bulk cultures in G418. Duplicate cultures were established in medium without G418 and grown for at least 2 weeks before use. For transient transfection of cell lines, the Lonza/Amaxa nucleoporation method was used. HMECs were transfected in monolayer culture using the Xfect Transfection Reagent (Clontech, #631317) with the suggested protocol. Transfected HMEC cultures were placed in medium containing 150 μg/ml G418 at 2 days after transfection and maintained for 12-14 days in the drug, a time sufficient to kill nontransfected cells. Cultures were placed in medium without G418 and harvested after one week of growth. Transfection efficiency was monitored using the pmaxGFP plasmid (Lonza/Amaxa) encoding a green fluorescent protein (GFP). Transfection efficiency was quantitated by flow cytometry using a FACSCalibur (Becton Dickinson Biosciences) with procedures described previously [29].

***Oligonucleotide “Pull-Down” Assays***

Biotinylated oligonucleotide and complement strand were heated to 95°C, annealed by slowly cooling to room temperature, and kept at 4°C until ready to use. Annealed oligonucleotides were mixed with nuclear extracts in 1X binding buffer (10 mM Tris pH 7.5, 8 mM NaCl, 10 μg/ml poly dI-dC, 1 mM EDTA, 0.1 mM β-mercaptoethanol, 4% glycerol) for 30 min at room temperature. Streptavidin beads were added and incubated with rocking at 4°C for 4 hrs. Beads were washed 5 times and the protein-oligonucleotide complexes were eluted using Laemlli buffer at 80°C for 5 min. Eluates were run on SDS-PAGE gel and examined by immunoblot analysis for Jun protein, using a rabbit polyclonal pan-Jun antibody (Santa Cruz Biotechnology, Inc, Santa Cruz, CA, #sc-44), and for cdk6 or cdk4, using mouse monoclonal antibodies (abcam #ab54576 and #ab75511, respectively). Cdk6 and cdk4 levels in whole cell and nuclear extracts were compared by immunoblot analysis and gel loading was normalized using β-actin and HDAC2 for whole cell and nuclear extracts, respectively. HDAC2 is a predominantly nuclear protein whose level is not detectably altered after transfection with cdk sequences [12].

***Immunohistochemistry***

Cells were washed with phosphate buffered saline and then incubated for 10 min with 10% neutral buffered formalin (Sigma-Aldrich, St. Louis, MO) and for 10 min with 0.5% Triton X-100 (Sigma-Aldrich, St. Louis, MO) in PBS. After incubation for 20 min in 2.5% horse serum (Vector, Burlingame, CA), immunohistochemistry was performed using anti-mouse Ig ImmPRESS and ImmPACT DAB (diaminobenzidine) peroxidase substrate kits (Vector) according to the manufacturer’s specifications with the mouse monoclonal antibodies listed in Table S5B. Incubations with specific antibodies (to cdk4, cdk6 or AKR1C3) were for 3 hrs at room temperature with antibodies diluted in 2.5% horse serum. Cells were counterstained with hematoxylin QS (Vector). Comparisons of each molecule in sets of cell lines were performed with identical antibody dilutions and photographs were taken and processed with the same settings, using a BX40 microscope with Q-Color3 digital camera (Olympus, Center Valley, PA) and Q Capture (QImaging, Surrey, BC Canada) and Adobe Photoshop CS (Adobe, San Jose, CA) software.
